# Translational Potential of Baicalein in Mitigating RSL3-Induced Ferroptosis in Fibroblasts: Implications for Therapeutic Interventions

**DOI:** 10.7150/ijms.91940

**Published:** 2024-05-13

**Authors:** Pao-Jen Kuo, Cheng-Shyuan Rau, Yi-Chan Wu, Chia-Wen Tsai, Chia-Jung Wu, Chia-Wei Lin, Ching-Hua Hsieh

**Affiliations:** 1 Department of Plastic Surgery, Kaohsiung Chang Gung Memorial Hospital and Chang Gung University College of Medicine, Kaohsiung, 83301, Taiwan.; 2 Department of Neurosurgery, Kaohsiung Chang Gung Memorial Hospital and Chang Gung University College of Medicine, Kaohsiung, 83301, Taiwan.

**Keywords:** Baicalein, Rat sarcoma virus selective lethal 3 (RSL3), Ferroptosis, Fibroblasts, Nuclear factor erythroid 2-related factor

## Abstract

**Background:** Ferroptosis is an iron-driven cell-death mechanism that plays a central role in various diseases. Recent studies have suggested that baicalein inhibits ferroptosis, making it a promising therapeutic candidate.

**Materials and Methods:** Fibroblast cultures were treated with different agents to determine the effects of baicalein on ferroptosis. Ferroptosis-related gene expression, lipid peroxidation, and post-treatment cellular structural changes were measured using real-time quantitative polymerase chain reaction, C11-BODIPY dye, and transmission electron microscopy, respectively.

**Results:** Baicalein significantly inhibited rat sarcoma virus selective lethal 3-induced ferroptosis in fibroblasts. Moreover, in baicalein-treated groups, reduced ferroptosis-related gene expression, decreased lipid peroxidation, and maintained cell structure was observed when compared with those of the controls.

**Discussion:** The ability of baicalein to counteract RSL3-induced ferroptosis underscores its potential protective effects, especially in diseases characterized by oxidative stress and iron overload in fibroblasts.

**Conclusion:** Baicalein may serve as a potent therapeutic agent against conditions in which ferroptosis is harmful. The compound's efficacy in halting RSL3-triggered ferroptosis in fibroblasts paves the way for further *in vivo* experiments and clinical trials.

## Introduction

Ferroptosis is a recently discovered type of controlled cell death that is characterized by lipid peroxide and iron-dependent reactive oxygen species buildup, which can lead to oxidative damage to cellular membranes 1-3. Moreover, ferroptosis is characterized by distinct morphological changes, including decreased mitochondrial volume, increased mitochondrial membrane density, and the absence of typical apoptotic features 4. Dysregulation of ferroptosis has been linked to several illnesses including neurological conditions 5-7, cardiovascular diseases 8, 9, acute and chronic kidney injury 10-12, ischemia-reperfusion injury 13, 14, and cancer therapy 15-17, making it an active research topic in cell biology and medicine.

The gene expression of these factors can be influenced by various signaling pathways and transcriptional regulators involved in ferroptosis. These include nuclear factor erythroid 2-related factor 2 (Nrf2) 18-21, the ferroptosis suppressor protein glutathione peroxidase 4 (GPX4) 22-24, the tumor suppressor protein p53 25-27, and System xc-, a cystine-glutamate antiporter consisting of solute carrier (SLC)3A2 and SLC7A11, which is crucial for importing cystine into the cell in exchange for glutamate 28, 29. The small-molecule compound rat sarcoma virus-selective lethal 3 (RSL3) is frequently employed as a ferroptosis activator, inhibits the function of GPX4, and causes a buildup of lipid peroxides, which initiates a chain reaction of lipid peroxidation. 30, 31. Lipid peroxidation disrupts the fluidity and integrity of the lipid bilayer, leading to membrane destabilization, permeabilization, cellular dysfunction, and programmed cells 30, 31.

Fibroblasts are a type of connective tissue cell and play essential roles in maintaining tissue structure, wound healing, and tissue repair. The induction of ferroptosis in fibroblasts has significant implications for various biological processes and pathological conditions, including tissue homeostasis, tissue fibrosis, wound healing, oxidative stress, and therapeutic interventions in the tumor microenvironment 32-38, thus making it a topic of intense interest in cell biology and biomedical research.

Baicalein is a flavonoid with a rich history in traditional medicine owing to its antioxidative and anti-inflammatory properties. Hence, it was considered as a modern therapeutic agent in this study. An inquiry aimed at screening a library of natural chemicals as potential ferroptosis inhibitors revealed that baicalein has potent inhibitory effects against erastin-induced ferroptosis in pancreatic cancer cells 39. Traditional Chinese and Japanese herbal medicines have long used baicalein, a natural flavonoid found in the roots of *Scutellaria baicalensis* and *Scutellaria lateriflora*, to treat bacterial and viral illnesses 40. Furthermore, baicalein exhibits antioxidative effects by scavenging reactive oxygen species, inhibiting lipid peroxidation, and attenuating oxidative damage associated with ferroptosis 41-45.

Although baicalein has shown promising potential for reducing ferroptosis in experimental studies, its effect on fibroblast ferroptosis is not well understood. This study aimed to determine the effects of baicalein on RSL3-induced ferroptosis in primary human fibroblasts. This study serves as a crucial link between basic cellular studies and clinical applications and lays the groundwork for subsequent *in vivo* studies and clinical trials. This approach is pivotal in moving beyond the theoretical approach and showcases a translational treatment with potential as a real-world medical solution.

## Materials and Methods

### Chemicals and reagents

Human skin fibroblasts were sourced from the Bioresource Collection and Research Center, Taiwan, under catalog number CG1639. Dulbecco's modified Eagle medium (DMEM) and Hanks' balanced salt solution (HBSS) were obtained from GIBCO (Grand Island, NY, USA). Ferroptosis inducers RSL3, baicalein and ferrostatin-1, were obtained from Sigma-Aldrich (St. Louis, MO, USA). The Kelch-like epichlorohydrin-associated protein 1 (Keap1)-Nrf2-antioxidant responsive element (ARE) antibody panel, cataloged under ARG30345, was sourced from Arigo Biolaboratories Corp (Hsinchu City, Taiwan). CytoScan water-soluble tetrazolium salt (WST-1) Cell Proliferation Assay kit (catalog number ab65475) was purchased from Abcam (Cambridge, UK). Horseradish peroxidase (catalogue number: AP132P) was purchased from Millipore (Burlington, MA, USA). The RNeasy Mini Kit (catalog number: 217004) was purchased from Qiagen (Hilden, Germany). Both the High-Capacity complementary (cDNA) Reverse Transcription Kit (catalog 4368814) and Power SYBR Green PCR Master Mix (catalog 4367659) were procured from Applied Biosystems (Foster City, CA, USA). Finally, C11-BODIPY 581/591 (catalog number D3861) was purchased from Thermo Fisher Scientific (Waltham, MA, USA).

### Cell viability

Cell viability was measured using the CytoScan WST-1 Cell Proliferation Assay, in which metabolically active cells reduce WST-1, resulting in the production of a colored formazan product. The number of viable cells was determined based on color intensity, which directly corresponded to cell viability. Briefly, fibroblasts were seeded into transparent 96-well microplates, with each well containing 10,000 cells. The cells were allowed to grow for a 24-h period in a moist environment with 5% CO_2_. These fibroblasts were then reconstituted in DMEM devoid of serum but enriched with 10% fetal bovine serum and 1% penicillin/streptomycin. Subsequent treatments involved introducing RSL3 in concentrations ranging from 0-2.0 μM and baicalein in dosages spanning from 0-1000 μM. The cells were allowed to grow and multiply, and after 0-16 h following RSL3 treatment, the cell culture medium was replaced with the WST-1 reagent. A microplate reader (Thermo Fisher Scientific) with a spectrophotometric function set at a wavelength of 450-490 nm, was used to measure formazan absorbance. Experiments were performed with six samples per group.

### Determination of Keap1/Nrf2/NQO1 & HO-1 activation

Western blotting was used to determine the activation of the Keap1/Nrf2/nicotinamide adenine dinucleotide phosphate hydrogen quinone dehydrogenase 1 (NQO1) and heme oxygenase 1 (HO-1) pathways. Fibroblast cytosolic proteins, which were either treated with phosphate-buffered saline (PBS) as a control or exposed to 0.5 μM RSL3 with or without 100 μM baicalein for 24 h, were isolated. The proteins were electrophoretically separated on polyacrylamide gels and transferred onto polyvinylidene fluoride membranes. These membranes underwent an overnight incubation at 4 °C with primary antibodies targeting Keap1, Nrf2, NQO1, and HO-1, sourced from the Keap1-Nrf2-ARE antibody panel kit. Primary antibodies were used at dilutions ranging from 1:500 to 1:1000. Following this, membranes were thoroughly washed in a 0.1% Tris-buffered saline/Tween 20 solution, maintained at 37 °C. Thereafter, the membranes were incubated for 2 h with a secondary antibody conjugated to horseradish peroxidase. The fluorescent signals were visualized and quantified using a FluorChem SP imaging device (Alpha Innotech, San Leandro, CA, USA). Experiments were performed with six samples per group.

### Determination of ferroptosis-related gene expression

Real-time quantitative polymerase chain reaction (PCR) was used to measure the expression of ferroptosis-related genes. Fibroblasts (1 × 10^5^) were cultured in a 10-cm dish and exposed to PBS (control group) and to 0.5 μM RSL3 in the presence or absence of baicalein (50 or 100 μM) or 10 μM ferrostatin-1 for 24 h. The collected cells were subjected to total RNA extraction using RNeasy Mini Kits, and the concentration of RNA was determined using an SSP-3000 NanoDrop spectrophotometer (Infinigen Biotech, City of Industry, CA, USA). The extracted RNA was subsequently transcribed into cDNA using a high-capacity cDNA Reverse Transcription kit. To amplify the cDNA, The Power SYBR Green PCR Master Mix was used to amplify the cDNA along with specific designed primers, including cyclooxygenase-2 (COX-2; forward: 5'-ACGATCCCTCCCTTACCATCAAA-3'; reverse: 5'-TCGGGAGTACTACTCGATTGTCAACG-3'); acyl-CoA synthetase long-chain family member 4 (ACSL4; forward: 5'-GTGGTTCTACTGGCCGACCTAAG-3'; reverse: 5'-CTGCAGCCATAGGTAAAGCAAGATATCTC-3'); prostaglandin-endoperoxide synthase 2 (PTGS2; forward: 5'-AGGGTTGCTGGTGGTAGGAAT-3'; reverse: 5'-TAGAGTGCTTCCAACTCTGCAGACA-3'); SLC3A2 (forward: 5'-GACTTGCTGTTGACTAGCTCATACCT-3'; reverse: 5'-GGAGAAGTTGAGCCGGCAAGA-3'); SLC7A11 (forward: 5'-CCAGATATGCATCGTCCTTTCAAGGT-3'; reverse: 5'-ATAATACGCAGGGACTCCAGTCAG-3'); GPX4 (forward: 5'-TAACGAAGAGATCAAAGAGTTCGCCG-3'; reverse: 5'-GGTGAAGTTCCACTTGATGGCATT-3'); and ferritin heavy chain 1 (FTH1; forward: 5'-CTTACTACTTTGACCGCGATGATGTG-3'; reverse: 5'-CACTCCATTGCATTCAGCCCG-3'). The primers for mouse glyceraldehyde 3-phosphate dehydrogenase as an internal control were 5'-GGAGAGTGTTTCCTCGTCCC-3' (forward) and 5'- ATGAAGGGGTCGTTGATGGC-3' (reverse). The Power SYBR Green PCR Master Mix with specific primers was used for cDNA amplification. The expression of ferroptosis-related genes was quantified using the 2-Ct method with normalized cycle threshold (Ct) values. All data were presented as the average ± standard deviation. To analyze the differences between group averages, one-way analysis of variance was utilized, followed by a post-hoc test using Fisher's least significant difference method. Experiments were performed with six samples per group. Statistical significance was set at P < 0.05.

### Determination of cell perioxidation

C11-BODIPY is a lipophilic fluorescent dye that is selectively incorporated into cellular membranes, making it useful for monitoring lipid peroxidation and oxidative stress. When C11-BODIPY is incorporated into membranes, its fluorescence emission spectrum shifts depending on its oxidation state. In an unoxidized state, it emits green fluorescence at a wavelength of approximately 581 nm, whereas upon oxidation, it undergoes a shift and emits red fluorescence at approximately 591 nm. This allows for monitoring of lipid peroxidation and oxidative stress in cells and tissues. After the specified treatment duration, cells were rinsed with PBS, collected using trypsin, and then suspended in 500 μl of HBSS with 2 μl of C11-BODIPY 581/591. This mixture was allowed to incubate for 15 min at 37 °C inside a tissue culture incubator. Post incubation, cells were cleaned using HBSS, spun down at 3,000 × g for a span of 5 min, and subsequently suspended in 200 μl of PBS. For each replicate, at least 10,000 events were gathered and evaluated using a flow cytometer (Becton Dickinson, Rutherford, NJ, USA) at a wavelength of 488 nm. Experiments were performed with six samples per group.

### Transmission electron microscopy (TEM) analysis

Ultrastructural modifications of the cells were analyzed using an HT-7700 transmission electron microscope (Hitachi, Tokyo, Japan). In the procedure, fibroblasts were either treated with PBS as a control or exposed to 0.5 μM RSL3, with or without the addition of 100 μM baicalein for 24 h. Following treatment, the cells were washed twice with PBS, centrifuged for collection, and immersed in agar. Once the agar solidified, cell aggregates were fixed using 2.5% glutaraldehyde in 0.1% sodium chloride buffer. After rinsing, dehydration, and embedding, samples were sectioned and examined under a transmission electron microscope at 100 kV.

## Results

### Baicalein treatment reduced Nrf2/HO-1 activation caused by RSL3 stimulation

**Figure 1:**
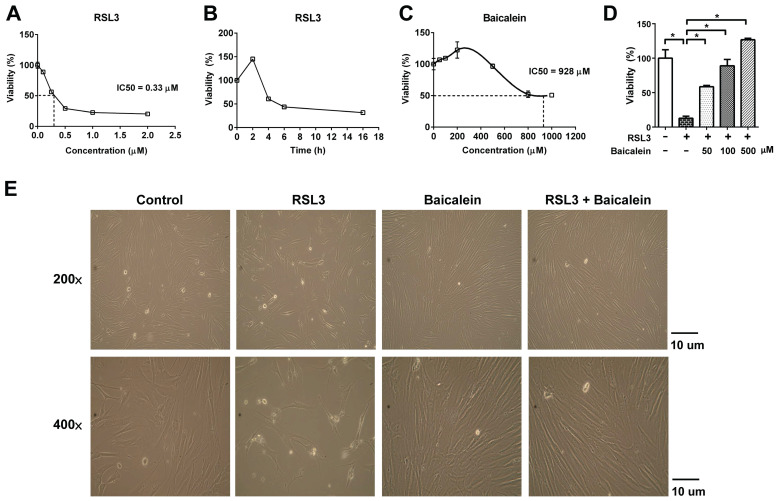
** Cell viability of fibroblasts.** Cell viability was detected using CytoScan WST-1 Cell Proliferation Assay upon treatment with (A) 0.5-2.0 μM RSL3 for 16 h; (B) 0.5 μM RSL3 for 0-16 h; or (C) 0-1000 μM baicalein for 16 h. IC_50_ indicates the half maximal inhibitory concentration; (D) The cell viability at 24 h later following additional of 50, 100, or 500 μM baicalein in the fibroblastes treated by 0.5 μM RSL3 for 16 h; (E) Morphology of fibroblasts under optic microscopy at magnifications of 200 and 400 × in the absence or presence of 0.5μM RSL3 treatment for 16 h, with or without subsequent 100 μM baicalein therapy for 24 h. WST-1: water-soluble tetrazolium salt; RSL3: rat sarcoma virus selective lethal 3.

**Figure 2:**
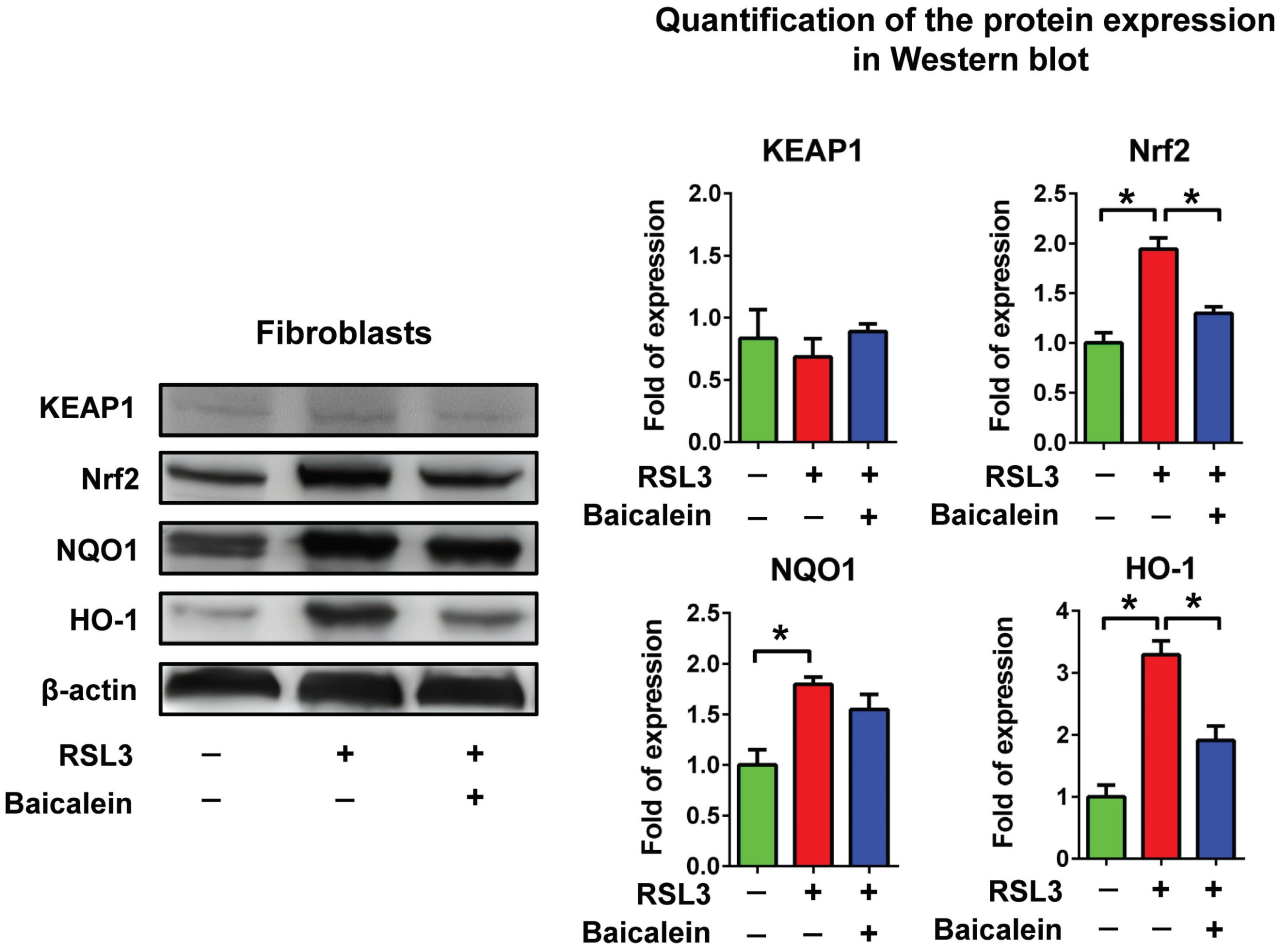
** Protein expression of Keap1/Nrf2/NQO1 & HO-1.** Detection of Keap1/Nrf2/NQO1 & HO-1 activation using western blotting in fibroblasts treated with PBS as a control or treated with 0.5 μM RSL3 in the presence or absence of 100 μM baicalein for 24 h. * Indicates a significant change (P < 0.05) in the samples (n = 6). The error bar represents the standard error of mean. Keap1: Kelch-like epichlorohydrin-associated protein 1; Nrf2: nuclear factor erythroid 2-related factor 2; NQO1: nicotinamide adenine dinucleotide phosphate hydrogen quinone dehydrogenase 1; HO-1: heme oxygenase 1; PBS: phosphate-buffered saline; RSL3: rat sarcoma virus selective lethal 3.

RSL3 treatment of fibroblasts for 16 h caused a substantial dose-dependent reduction in cell viability, with a half-maximal inhibitory concentration (IC_50_) of 0.33 μM (Figure 1A). Cell viability of the fibroblasts treated with 0.5 μM RSL3 significantly decreased at 4 h and persisted till at least at 16 h (Figure 1B). Additionally, the 500 μM baicalein treatment had no discernible effect on fibroblast cell vitality. The IC_50_ of baicalein treatment for fibroblasts was 928 μM (Figure 1C). Baicalein at concentrations of 50, 100, and 500 μM improved fibroblast vitality at 24 h later following 0.5 μM RSL3 therapy (Figure 1D). Except for the more dispersed distribution of the cells, there was no discernible morphological change in each cell under light microscopy (Figure 1E). As shown in Figure 2, the expression of Nrf2, as well as downstream NQO1 and HO-1, significantly increased after the fibroblasts 0.5 μM RSL3 treatment for 16 h. Activation of HO-1 and Nrf2 expression was significantly lowered by the addition of 100 μM baicalein; however, the upregulation of NQO1 by RSL3 treatment was not significantly reduced by the addition of baicalein. The expression of Keap1 was not significantly altered following RSL3 treatment in the presence or absence of baicalein.

### Expression of ferroptosis-related genes and cell perioxidation

**Figure 3:**
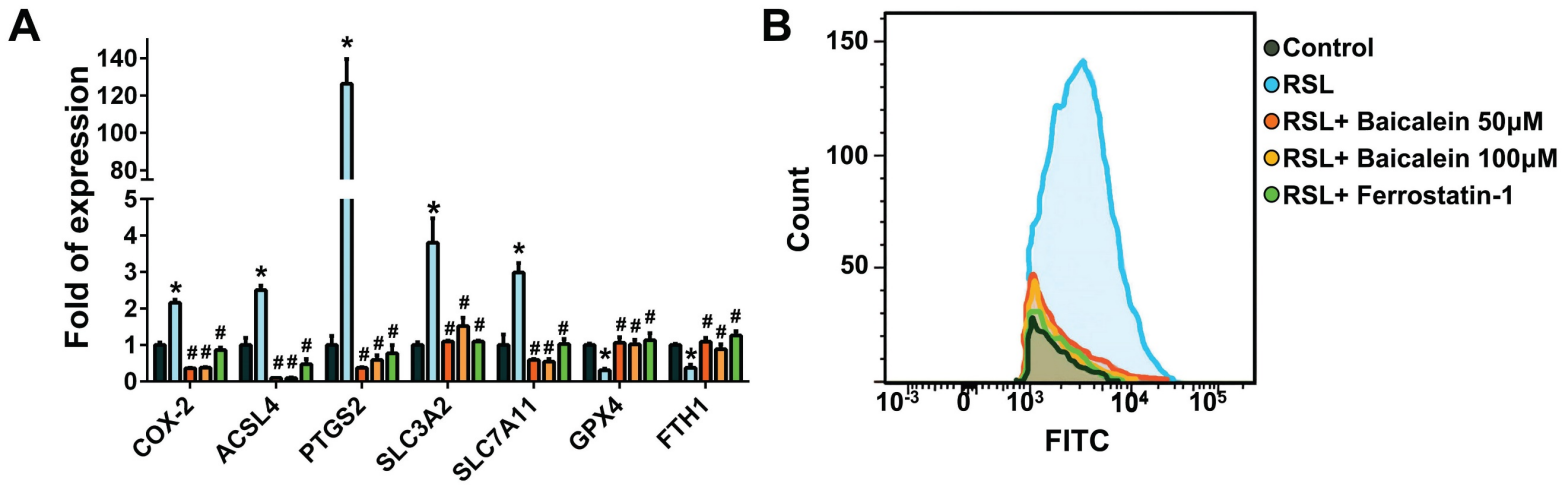
** Expression of ferroptosis-related genes and perioxidation.** (A) Real-time quantitative polymerase chain reaction of ferroptosis-related genes in 1 × 10^5^ fibroblasts exposed to PBS (control) or to 0.5 μM RSL3 in the presence or absence of 50 or 100 μM baicalein or 10 μM ferrostatin-1 for 24 h. * Significant change (P < 0.05) compared with the control values (n = 6). # Significant change (P < 0.05) compared with those receiving RSL3 treatment. The error bar represents the standard error of mean. (B) Expression of C11-BODIPY detected using flow cytometry in those fibroblasts under the same conditions listed above. PBS: phosphate-buffered saline; RSL3: rat sarcoma virus selective lethal 3.

Figure 3A demonstrates that when treated with 0.5 μM RSL3 for 16 h, there was an increase in the expression of ferroptosis-associated genes such as COX-2, ACSL4, PTGS2, SLC3A2, and SLC7A11. Concurrently, there was a reduction in GPX4 and FTH1 levels, which are typically reduced during ferroptosis. Moreover, the addition of 50 and 100 μM baicalein, or 10 μM ferrostatin-1 significantly reduced the over-expression of COX-2 and increased the down-regulated GPX4 and FTH1 levels. Flow cytometry detecting C11-BODIPY revealed that RSL3 treatment increased cell peroxidation, which was significantly reduced by adding 50 and 100 μM baicalein, or 10 μM ferrostatin-1 (Figure 3B).

### TEM analysis

**Figure 4:**
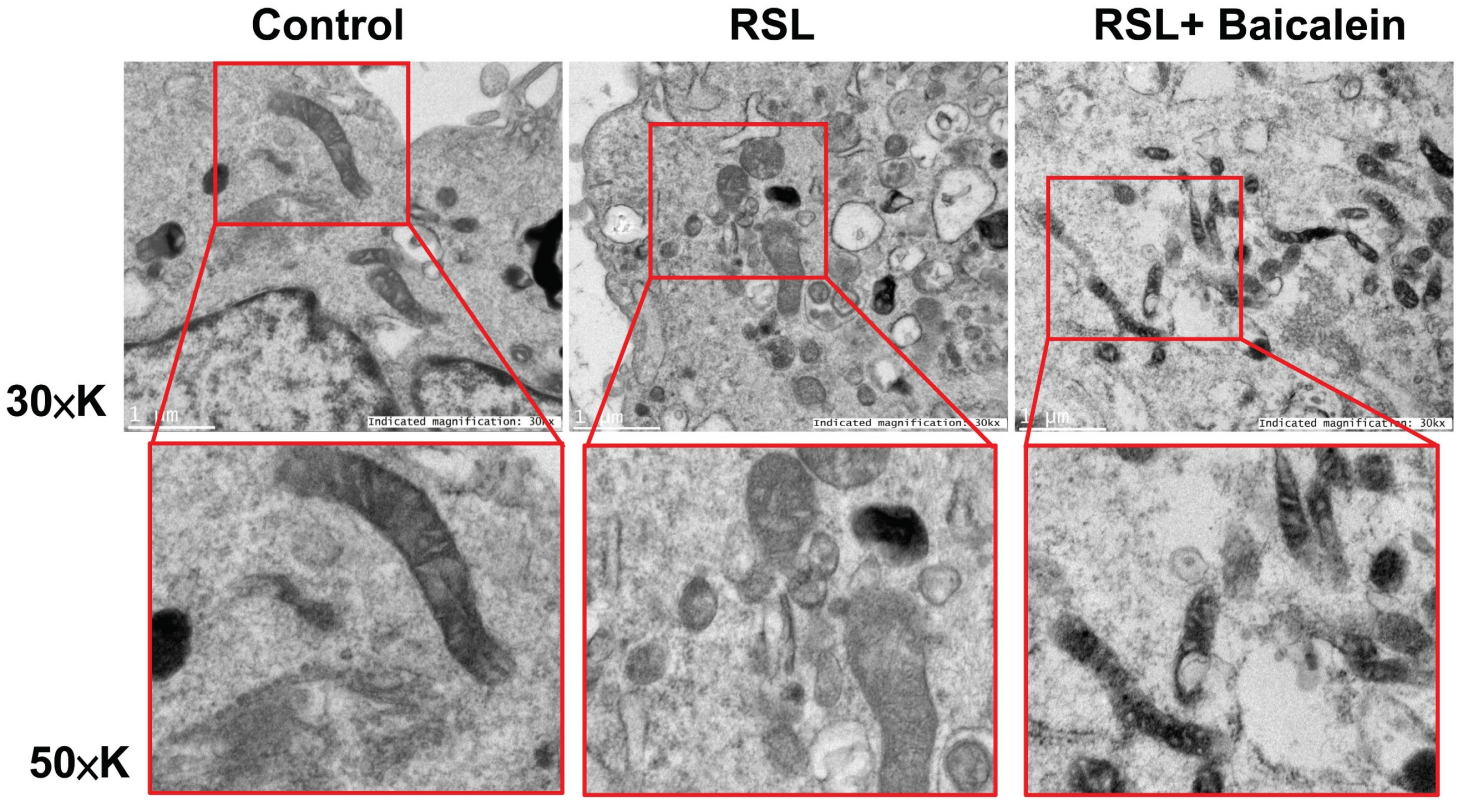
** Representative images of the mitochondrial morphology.** Mitochondrial shrinkage with decreased cristae and increased membrane density characterized the typical morphology of cells undergoing ferroptosis. Magnification 30×K (top row), 50×K (bottom row).

The mitochondrial ultrastructure of the fibroblasts treated with 0.5 μM RSL3 in the presence or absence of 100 μM baicalein for 24 h was detected using TEM. Cells treated with PBS were used as controls. Ferroptotic cells typically had smaller-than-normal mitochondria, dense mitochondrial membranes, few cristae, and ruptured outer mitochondrial membranes (Figure 4A). These characteristics were diminished after 100 μM baicalein treatment.

## Discussion

Keap1 detects oxidative and electrophilic disturbances in the cytoplasm. When confronted with oxidative stress or electrophiles, Keap1 undergoes changes that cause Nrf2 to detach from it. Consequently, Nrf2 becomes stable and accumulates in the cytoplasm. Subsequently, Nrf2 moves to the nucleus and attaches to DNA sequences called AREs 46-48. This connection initiates the transcription of several protective genes such HO-1 46-48 and NQO1 49-51. HO-1 is vital for protection against oxidative stress and for ensuring cellular balance. The protein breaks down haem, which is found in hemoglobin and similar proteins, into biliverdin, iron, and carbon monoxide. Biliverdin is transformed into bilirubin, which is known for its antioxidant benefits 46-48. In contrast, NQO1 aids in detoxifying quinones, thereby helping to shield cells from oxidative harm and potential carcinogenic effects 49-51.

Numerous studies have demonstrated the antiferroptotic capabilities of baicalein 41-45. In rat cardiomyocytes, baicalein countered ferroptosis triggered by ischemia/reperfusion by limiting the build-up of reactive oxygen species and malondialdehyde 42. Baicalein also protected against RSL3-induced ferroptosis in melanocytes, primarily by boosting GPX4 levels 43. In mice, targeting ferroptosis using baicalein led to improved survival rates after total-body irradiation 45. Notably, baicalein effectively halts ferroptosis through 12/15-lipoxygenases-driven lipid peroxidation, presenting neuroprotective effects in cases such as post-trauma FeCl3-induced epileptic seizures post-trauma 44 and traumatic brain injuries 41, 52. Notably, baicalein's anti-ferroptosis process surpasses that of established ferroptosis inhibitors such as ferrostatin-1 and liproxstatin-1 53. Recent findings have also highlighted RSL3's role in inducing ferroptosis, as evidenced by the disruption of associated genes, mitochondrial dysfunction, and escalated cell peroxidation in cultured fibroblasts. Additionally, baicalein curtails RSL3-driven ferroptosis, and its anti-ferroptosis effect is linked to activation of the Nrf2/HO-1 signaling pathway. This is achievable even with other recognized inhibitors such as ferrostatin-1. Therefore, baicalein holds promise as a treatment for cancer 36, 54 or liver fibrosis, in which fibroblasts significantly influence disease progression 55.

In the evolving landscape of biomedical research, ferroptosis has emerged as a critical area with substantial implications for various clinical conditions 56, 57, including cancer, neurodegeneration, and ischemic injury. The current research, which focuses on the inhibition of RSL3-induced ferroptosis in fibroblasts using baicalein, positions itself at the forefront of translational medicine. This study bridges this significant gap by exploring the effects of baicalein in a novel context, being its interaction with fibroblasts under ferroptotic stress.

However, the pioneering exploration of the effects of baicalein on RSL3-induced ferroptosis in fibroblasts has limitations. Primarily, the study focuses exclusively on *in vitro* models, which, although informative, cannot fully replicate the complex *in vivo* environment. This limitation highlights the need for subsequent animal studies to validate these findings and explore their systemic effects. Additionally, the scope of the study was confined to a single cell type, warranting further research across different cell types and tissues to understand its broader implications. Furthermore, although this research provides valuable insights, it does not address the potential side effects or toxicity of baicalein at therapeutic doses, which are crucial for clinical translation. Moreover, while this is not the initial study to demonstrate that baicalein inhibits cellular ferroptosis for therapeutic purposes, the results will contribute to the existing body of knowledge in this field. Nevertheless, additional research into the underlying mechanism is necessary to fully exploit the therapeutic capabilities of baicalein.

## Conclusion

The findings of this study highlight the protective role of baicalein against RSL3-induced ferroptosis, thereby offering promising insights for drug development. Moreover, the study suggests potential avenues for the treatment of diseases in which ferroptosis plays a pivotal role.

## Abbreviations

RSL3: rat sarcoma virus selective lethal 3
Nrf2: nuclear factor erythroid 2-related factor 2
GPX4: glutathione peroxidase 4
PCR: polymerase chain reaction
SLC: solute carrier
DMEM: Dulbecco's modified Eagle medium
HBSS: Hanks' balanced salt solution
Keap1: Kelch-like epichlorohydrin-associated protein 1
ARE: antioxidant responsive element
cDNA: complementary
WST-1: water-soluble tetrazolium salt
NQO1: nicotinamide adenine dinucleotide phosphate hydrogen quinone dehydrogenase 1
HO-1: heme oxygenase 1
PBS: phosphate-buffered saline
COX-2: cyclooxygenase-2
ACSL4: acyl-CoA synthetase long-chain family member 4
PTGS2: prostaglandin-endoperoxide synthase 2
FTH1: ferritin heavy chain 1
TEM: transmission electron microscopy
IC_50_: half-maximal inhibitory concentration
